# Progressive Familial Intrahepatic Cholestasis Type 3 Homozygous Pathogenic Variant c.2906G>A in the ATP Binding Cassette Subfamily B Member 4 (ABCB4) Gene: A Case Report of an Unusual Presentation

**DOI:** 10.7759/cureus.32455

**Published:** 2022-12-12

**Authors:** Badriah G Alasmari, Syed Rayees, Mohammed Alomari, Lina Elzubair, Yassin Hamid

**Affiliations:** 1 Pediatrics, Armed Forces Hospitals Southern Region (AFHSR), Khamis Mushait, SAU; 2 Pathology, Armed Forces Hospitals Southern Region (AFHSR), Khamis Mushait, SAU; 3 Pediatrics, Neleen University, Khartoum, SDN

**Keywords:** a new variant, a case report, epstein-barr virus infection, abcb4 gene, progressive familial intrahepatic cholestasis

## Abstract

Progressive familial intrahepatic cholestasis (PFIC) describes a heterogeneous group of autosomal-recessive childhood liver disorders in which cholestasis of hepatocellular origin frequently manifests during infancy or the first year of life and progresses to liver failure. We report a case of a five-year-old boy with homozygous pathogenic variant c.2906G>A in the ATP binding cassette subfamily B member 4 (*ABCB4)* gene presented with hepatosplenomegaly and cytopenia without a history of jaundice or itching; he had a history of Epstein-Barr virus infection and family history of liver disease. The patient was started on ursodeoxycholic acid and fat-soluble vitamins and referred to a liver transplant center.

## Introduction

ATP-binding cassette subfamily B member 4 (*ABCB4*) gene mutations impair bile production and cause cholestasis of hepatocellular origin in patients with progressive familial intrahepatic cholestasis (PFIC), an autosomal recessive condition [1]. The precise incidence is still unclear; however, it is thought to occur between 1/50,000 and 1/100,000 births [2]. In the hepatocellular transport system, three different kinds of PFIC have been shown to be mutated. While the development of PFIC type 3 might occur later in infancy, youth, or even during early adulthood [3], PFIC type 1 and PFIC type 2 often manifest in the first few months of life. Cholestasis, pruritus, and jaundice are the primary clinical symptoms. Cirrhosis and advanced liver disease are often diagnosed in PFIC patients before reaching maturity [1,2]. For patients with PFIC1 and PFIC2, the serum GGT activity is normal; however, for PFIC3 individuals, it is increased. Bile phospholipid secretion is compromised in PFIC-3, whereas it is decreased in PFIC-1 and PFIC-2 [4].

The *ABCB4* gene, also known as multidrug resistance protein 3 (MDR3), is situated on chromosome 7, and it is the source of the genetic deficiency that leads to PFIC3 [5]. The cholangiopathy PFIC3 is a real-world illustration of a canalicular transport abnormality. From neonatal cholestasis through cirrhosis in early adulthood, the PFIC3 phenotypic spectrum is wide [2]. About one-third of individuals have clinical indications of cholestasis during the first year of life, with PFIC3 kids seeing these signs more seldom during the neonatal period. The primary symptoms in adolescents and young adults are cirrhosis and gastrointestinal bleeding caused by portal hypertension, and pruritus is often minor [6]. Portal hypertension, prolonged icteric or anicteric cholestasis, and liver failure are symptoms of progression. At a mean age of seven-and-half years, liver transplantation is needed in half of all PFIC3 patients [1,2]. During adolescence, hepatocarcinoma may also manifest. Patients with PFIC3 have normal serum cholesterol levels, persistently high serum Gamma-glutamyl transferase (GGT) activity, and moderately elevated quantities of main bile salts in their blood [2].

## Case presentation

A five-year-old boy with consanguineous parents of Arab ethnicity was delivered at term through spontaneous vaginal delivery with no neonatal intensive care unit (NICU) admission. He has a family history of liver disease in his uncles, one of them died at the age of 11 years, and one uncle underwent a liver transplant and died at the age of 30 years. His parents did not know what disease they had. At the age of three years, his parents noticed he had abdominal distention and cough, so they sought advice at a hospital, where he was diagnosed with Epstein-Barr virus (EBV) with hepatosplenomegaly. They came to our hospital for a follow-up. He was initially seen in a pediatric gastroenterologist clinic.

**Figure 1:**
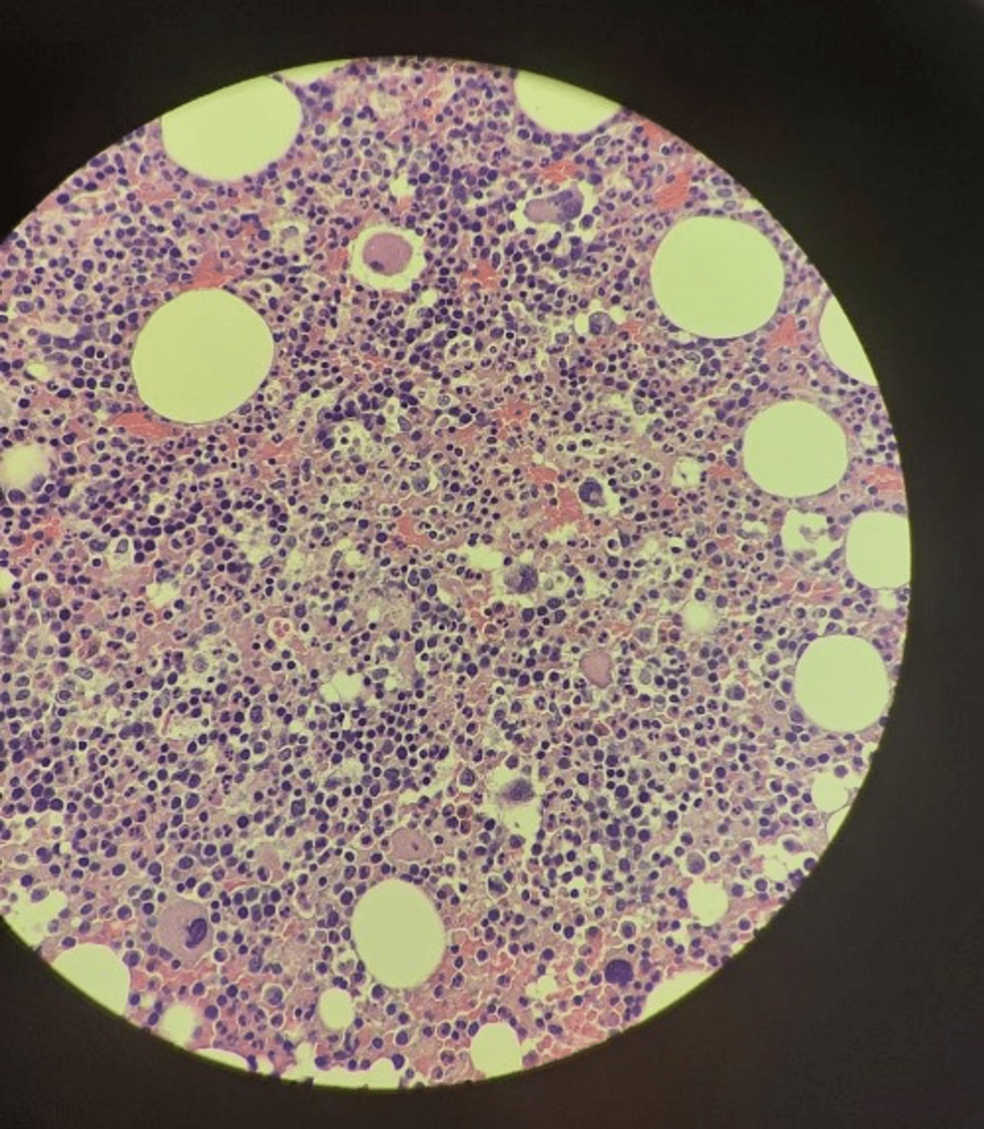
Bone marrow trephine biopsy showing hypercellular BM with trilineage hyperplasia and prominent juvenile megakaryocytes (10X, Hematoxylin and Eosin stain)

**Figure 2:**
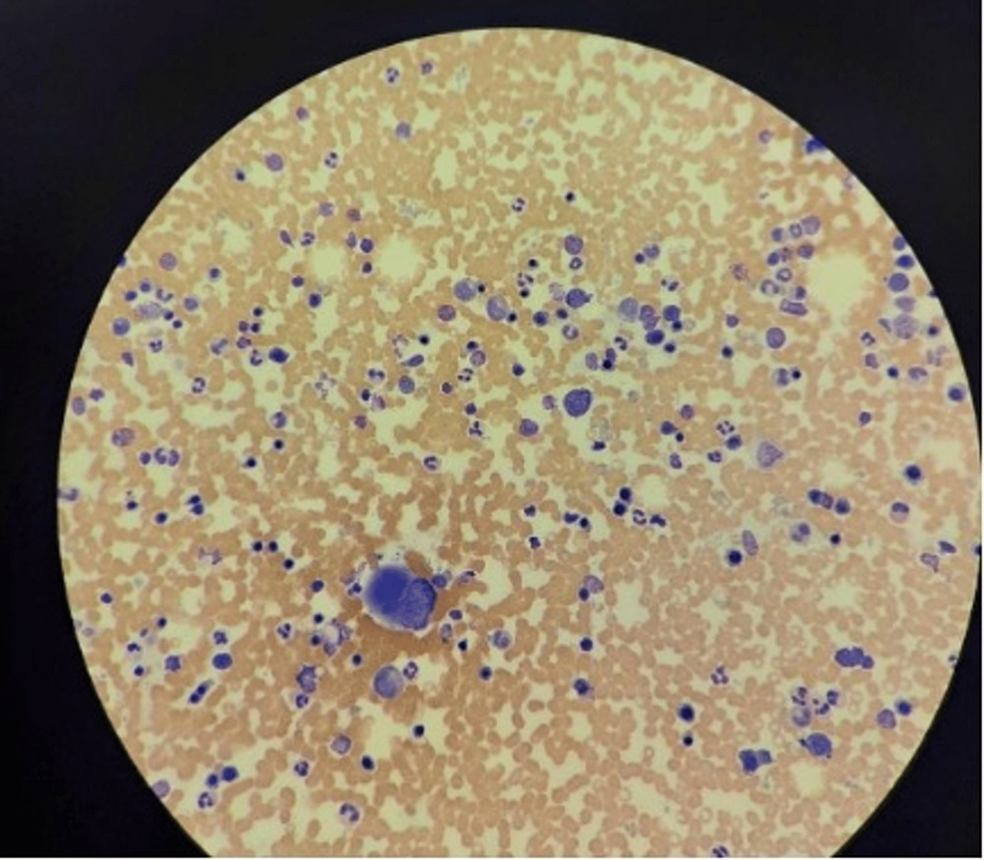
Bone marrow aspirate (x10) showing erythroid hyperplasia with micronormoblastic matutleation and two juvenile megakaryocytes

On physical examination: he has no dysmorphic features, is developmentally normal, chest clear, liver 4 cm below the costal margin, spleen 4 cm below the costal margin, no ascites, no complaints of itching, and passing a normal stool. The liver profile showed elevated liver enzyme, while complete blood count (CBC) values are almost within the normal range (Table 1). Bone marrow (BM) showed hypercellular BM with trilineage hyperplasia and prominent juvenile megakaryocyte (Figures 1, 2 ). Abdomen ultrasound scan showed hepatosplenomegaly with normal patent portal vein 9 mm. He was treated symptomatically for EBV.

**Table 1:** Laboratory’s results of liver enzymes and CBC of the patient

Measured entity	Current value	Normal range
Aminotransferase (ALT)	131 U/L	11-39 U/L
Aspartate aminotransferase (AST)	155 U/L	22-58 U/L
Gamma-glutamyl transferase (GGT)	73 U/L	5-40 U/L
White blood cells (WBC)	3.49 units 10^9L	4.5-13.5 units 10^9L
Platelets	101 units 10^9/L.	150-400 units 10^9/L
Hemoglobin (Hb)	11.1 g/l	10.9-15 g/l

During follow-up, the patient showed bi-cytopenia with persistent elevated liver enzymes and hepatosplenomegaly. On further workup, high-performance liquid chromatography (HPLC), normal, hepatic serology negative, brucella titer normal, EBV PCR 1110 copies/ml, Immunoglobulin G (IgG) positive, IgM negative, ferritin 29 microgram/L (normal10.3-55), alfa fetoprotein 1.3 ug/L (normal-0.8-4.5), lactate dehydrogenase (LDH) 179mmol/L (normal 140-304), smooth muscle antibody, liver kidney microsomes antibody and malarial parasite negative. Because of his strong family history, we sent a genetic study that identified the homozygous pathogenic variant c.2906G>A in the *ABCB4* gene. He was started on Ursodeoxycholic acid and fat-soluble vitamin supplementation.

Around two months later, he presented to the ER complaining of itching and epistaxis. On examination, no lymphadenopathy, non-icteric, chest equal air entry, normal heart sounds, abdomen soft lax non-tender liver palpable firm 4 cm below right costal margin and increased in spleen size around 7 cm, no ascites. Investigation shows WBC 3.49 units 10^9L (normal:4.5-13.5), Hb 10.7 g/l (normal-10.9-15), platelets 90 units 10^9/L, liver enzyme ALT 112 U/L(normal:11-39), AST 141 U/L (normal:22-58), GGT 82, total bilirubin 24 micromol/L (< 34.3), albumin 30g/L (35-50), direct bilirubin not available, INR 1.29 (normal <1.4). Ultrasound scan of his abdomen showed a liver (span 116mm), patent portal vein 12mm with normal flow pattern and direction, patent hepatic veins, spleen (span 126mm), contracted gall bladder, and common bile duct size 3 mm.

He was started on intravenous fluids, proton pump inhibitors, and vitamin k and treated symptomatically. Thereafter, his condition improved. As he was suffering from hepatosplenomegaly and hypersplenism and with a genetic diagnosis of progressive familial intrahepatic cholestasis type 3 (PFIC type 3), he was referred to a higher center hospital with an advanced hepatology unit with the facility of liver transplantation.

## Discussion

The uncommon hereditary condition known as progressive familial intrahepatic cholestasis (PFIC) covers a variety of different illnesses that all include bile acid secretion or transport abnormalities [7]. Before reaching adulthood, patients often proceed to end-stage liver disease after first presenting in childhood or infancy. Ursodeoxycholic acid, a therapeutic bile acid, may improve the clinical state of PFIC3 patients; however, it is often the only 3 of 4 successful treatments [8]. Two-thirds of PFIC cases are made up of PFIC1 and PFIC2, while one-third of cases are made up of PFIC3. However, more recently, genetic testing has emerged as the gold standard for diagnosing PFIC. Historically, the diagnosis of PFIC has been based on a mix of clinical and laboratory or biochemical techniques [7].

The multidrug resistance protein 3 (MDR3) is a glycoprotein, which is encoded by the *ABCB4* gene on chromosome 7q21, which may become mutated and lead to PFIC3, an autosomal recessive disease. 74 kb of the gene's 27 coding exons are used in protein synthesis. These individuals present with a broad range of symptoms, including end-stage liver disease, cirrhosis, gallstones, transitory newborn cholestasis, episodic cholestasis, and transient cholestasis [1].

From the neonatal era until maturity, the age of PFIC3 onset might be quite different. Jaundice, itchiness, variceal hemorrhage (portal hypertension), growth retardation, decreased bone density, and learning difficulties are some of the early-onset disease symptoms [1].

Our case never had jaundice, and the first presentation was with hepatosplenomegaly at the age of three, with no other significant clinical signs like jaundice, pruritis, and cholestasis. At that time, he was diagnosed with infectious mononucleosis (EBV positive) with pancytopenia and atypical lymphocytes. His initial clinical presentation can justify EBV. During his follow-up at our hospital, we found he did not have any resolution in his elevated liver enzymes and organomegaly. Autoimmune workup and viral serology were negative. A genetic study was sent, whose results show the homozygous pathogenic variant c.2906G>A in the *ABCB4* GENE. The patient was started on ursodeoxycholic acid and fat-soluble vitamins and referred to a liver transplant center.

## Conclusions

It can be concluded that as PFIC is an autosomal recessive disease, family history is the major diagnostic marker, and it can be confirmed by the genetic study. In this case, we found that PFIC TYPE 3 can have an unusual presentation, like hepatosplenomegaly, before the clinical picture of cholestasis. It is also shown in this case that the patient with PFIC TYPE 3 may not have the usual symptoms of jaundice, pruritis, and cholestasis.
